# Potential Activity of Subglacial Microbiota Transported to Anoxic River Delta Sediments

**DOI:** 10.1007/s00248-016-0926-2

**Published:** 2017-01-09

**Authors:** Karen A. Cameron, Marek Stibal, Nikoline S. Olsen, Andreas B. Mikkelsen, Bo Elberling, Carsten S. Jacobsen

**Affiliations:** 1 Department of Geochemistry, Geological Survey of Denmark and Greenland (GEUS), Øster Voldgade 10, DK-1350 Copenhagen, Denmark; 2 Center for Permafrost (CENPERM), University of Copenhagen, Øster Voldgade 10, DK-1350 Copenhagen, Denmark; 3 Institute of Biological, Environmental and Rural Sciences (IBERS), Aberystwyth University, Penglais, Aberystwyth, SY23 3FL UK; 4 Department of Ecology, Faculty of Science, Charles University, Viničná 7, 128 43 Prague, Czech Republic; 5 Department of Environmental Science, Aarhus University, Frederiksborgvej 399, DK-4000 Roskilde, Denmark

**Keywords:** Subglacial environment, River delta, Sulphate reduction, Methanogenesis, Meltwater export, Methane oxidation

## Abstract

The Watson River drains a portion of the SW Greenland ice sheet, transporting microbial communities from subglacial environments to a delta at the head of Søndre Strømfjord. This study investigates the potential activity and community shifts of glacial microbiota deposited and buried under layers of sediments within the river delta. A long-term (12-month) incubation experiment was established using Watson River delta sediment under anaerobic conditions, with and without CO_2_/H_2_ enrichment. Within CO_2_/H_2_-amended incubations, sulphate depletion and a shift in the microbial community to a 52% predominance of *Desulfosporosinus meridiei* by day 371 provides evidence for sulphate reduction. We found evidence of methanogenesis in CO_2_/H_2_-amended incubations within the first 5 months, with production rates of ~4 pmol g^−1^ d^−1^, which was likely performed by methanogenic *Methanomicrobiales*- and *Methanosarcinales*-related organisms. Later, a reduction in methane was observed to be paired with the depletion of sulphate, and we hypothesise that sulphate reduction out competed hydrogenotrophic methanogenesis. The structure and diversity of the original CO_2_/H_2_-amended incubation communities changed dramatically with a major shift in predominant community members and a decline in diversity and cell abundance. These results highlight the need for further investigations into the fate of subglacial microbiota within downstream environments.

**Electronic supplementary material:**

The online version of this article (doi:10.1007/s00248-016-0926-2) contains supplementary material, which is available to authorized users.

Greenland has been estimated to contribute towards ~7% of the world’s suspended sediment flux [1]. At Leverett Glacier, in the southwest of the Greenland Ice Sheet (GrIS), approximately 10^11^ microbial cells per cubic meter of meltwater are exported alongside these sediments [2]. These cells and sediments are mostly sourced from the subglacial environment [1, 2], and the Watson River channels them to the downstream fjord with minimal changes to their community composition [2]. Under the assumption that cells are deposited alongside glacial sediments in the river delta at the fjord head, which likely amass at rates of 9–16 mm yr^−1^ [1], and assuming that once buried under subsequent depositions the environment becomes waterlogged and anoxic, this study sought to consider the potential activity of transported subglacial communities, especially methanogens, whose potential activity and greenhouse gas production has been reported previously [3–8]. We hypothesised that subglacial methanogens resume their activity after deposition within anoxic sediments.

To test our hypothesis, we established long-term (12-month) anaerobic incubations using sediment core material sampled from the Watson River delta at the head of Søndre Strømfjord, where the meltwaters of four southwest GrIS outlet glaciers drain (Leverett, Russell, Ørkendalen and Isorlersuup glaciers). Incubations were set up at 2 °C in the dark, with and without CO_2_/H_2_ enrichment. We measured changes in methane and sulphate concentrations and assessed the temporal variability of bacterial and archaeal abundance and community structure in order to gain insight into anaerobic microbial activity. Details of the sample site, experimental set-up, gas and water sample analyses, qPCR set-up, sequencing protocol and sequence analysis are provided within Online Resource 1.

We observed an increase in methane within the CO_2_/H_2_-amended incubations, from near the limit of quantification 5.14 × 10^−4^ ± 0.22 × 10^−4^ μmol on day 0 to 30.34 × 10^−4^ ± 5.99 × 10^−4^ μmol on day 147 (Fig. 1a). This increase was significant (two-tailed *t* test; *t* = 7.27; *p* < 0.01) and the mean production rate during this period was 4.29 ± 1.02 pmol g^−1^ d^−1^ (wet weight), in line with rates measured from Russell Glacier subglacial sediments [6]. Between days 147 and 252, there was a significant decrease in methane to 12.60 × 10^−4^ ± 2.19 × 10^−4^ μmol (two-tailed *t* test; *t* = 4.81; *p* < 0.01), followed by another increase to 23.23 × 10^−4^ ± 3.00 × 10^−4^ μmol by day 371 (two-tailed *t* test; *t* = 4.97; *p* < 0.01; Fig. 1a). The decrease between days 147 and 252 was coincident with a significant depletion in sulphate from 1.49 ± 1.29 μmol to 0 ± 0 μmol (two-tailed *t* test; *t* = 25.99; *p* < 0.01; Fig. 1b). We calculate that between days 147 and 252, methane oxidation occurred at rates of 4.22 pmol g^−1^ d^−1^ and sulphate reduction occurred at rates 1000-fold greater, at 3.54 nmol g^−1^ d^−1^, assuming that sulphate was not depleted before day 252. Within control incubations, methane increased minimally between days 60 and 251 (~2.2 × 10^−4^ μmol increase; linear regression *R* ^2^ coefficient (LRR^2^) ≥ 0.83; *p* < 0.01). Methane abundance measurements from control and CO_2_/H_2_-amended incubations were statistically different on days 60, 251/252 and 371 (two-tailed *t* test; *t* = 3.64; *p* = 0.01). There was no significant difference between methane measured on day 0 under the two incubation conditions (two-tailed *t* test; *t* = 0.87; *p* = 0.48). Sulphate was found to decrease significantly between days 115 and 251 within the control set-up (LRR^2^ = 0.93; *p* < 0.01).

**Fig. 1:**
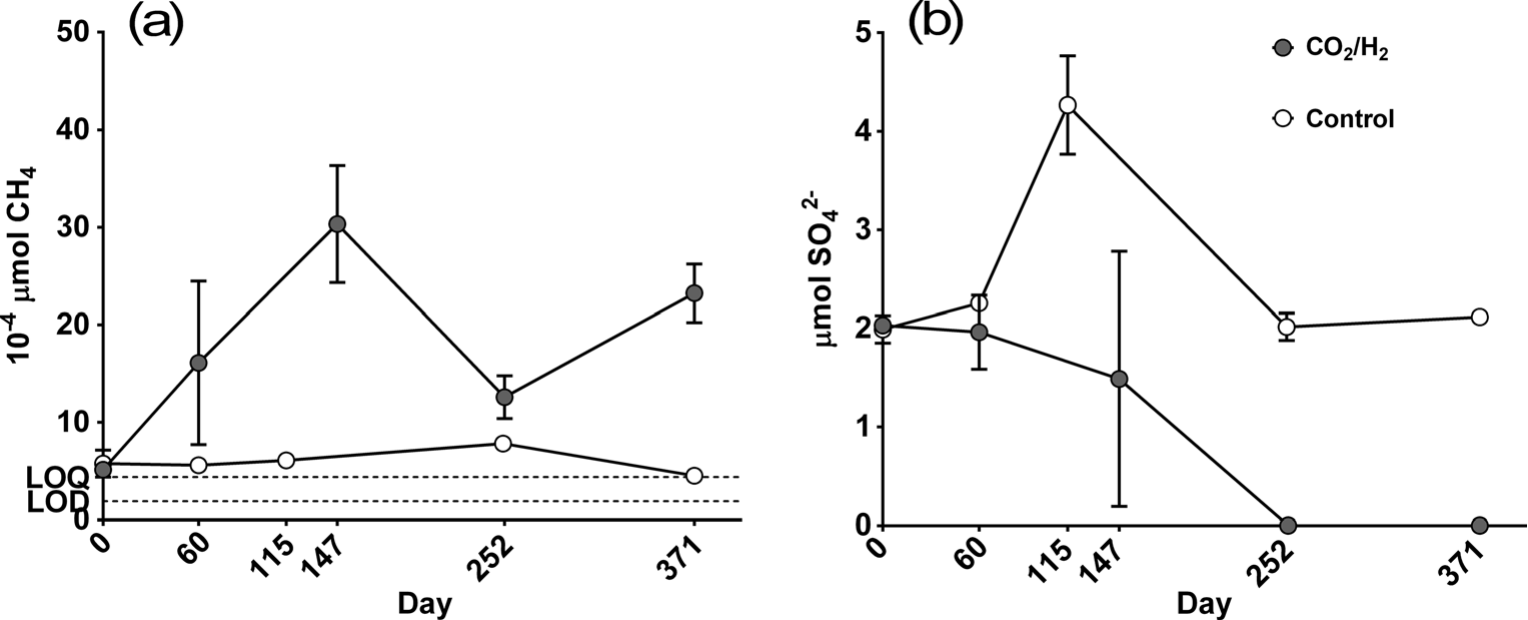
Chemistry of incubations. **a** Methane abundance per incubation (10^−4^ μmol). **b** Sulphate abundance per incubation (μmol). *Error bars* show the standard deviations. *Dashed lines* indicate the limit of detection (LOD) and limit of quantification (LOQ) for methane analysis. The LOD for sulphate analysis was 0.52 μmol L^−1^ or an absolute abundance of 0.0094 μmol within each incubation

Bacterial and archaeal cell abundance decreased threefold during the CO_2_/H_2_-amended incubations from 2.61 × 10^5^ ± 0.96 × 10^5^ cells per gram at day 0 to 7.67 × 10^4^ ± 1.20 × 10^4^ cells per gram at day 371. Catchall-calculated alpha diversity of 16S rRNA gene amplicons was notably and significantly reduced during the CO_2_/H_2_-amended experiment, from 1307 ± 65 at the start of the experiment to 268 ± 77 at the end (LRR^2^ = 0.78; *p* < 0.01). No significant change in diversity was observed within the control incubations (LRR^2^ = 0.27; *p* = 0.09). The CO_2_/H_2_-amended communities were found to be highly dissimilar to the control incubation communities when a two-way analysis of similarity (ANOSIM) was performed, accounting for differences in sampling time (*R* = 0.83; *p* < 0.01). Due to the reduction in cell abundance and diversity, and their ANOSIM-calculated uniqueness, we focus on the CO_2_/H_2_-amended communities herein.

CO_2_/H_2_-amended communities sampled at different time points were found to be dissimilar (*GlobalR* = 0.71; *p* < 0.01). The greatest dissimilarities were found between the day 0 communities and the other communities (*R* ≥ 0.82), while days 60, 147 and 371 communities were found to be moderately similar to each other (*R ≤* 0.44); however, due to a low number of samples, the results of these analyses were not statistically significant. SIMPER analysis highlighted 19 operational taxonomic units (sequence clusters with ≥97% similarity; OTU) that contributed towards ≥1% of the dissimilarities found between communities sampled at different time points (Fig. 2). Of these OTU, four that were closely related to *Desulfosporosinus meridiei* of the *Peptococcaceae* family dominated the communities on day 371 (Fig. 2d). *Desulfosporosinus* are sulphate reducers that grow chemolithoautotrophically on hydrogen [9]. In total, 17 OTU that were closely related to *D. meridiei* were found within the day 371 sampled communities, accounting for 4.00 × 10^4^ ± 0.31 × 10^4^ cells per gram, or 52% of the population. The establishment of these sulphate-reducing species, which correlated with the depletion of sulphate (Pearson’s *r* = −0.96; *p* = 0.04), is convincing of the potential for subglacially sourced microbes to reduce sulphate within delta sediments, consistent with previous studies from subglacial environments [4, 8], although the incubation bottle effect cannot be excluded.

**Fig. 2:**
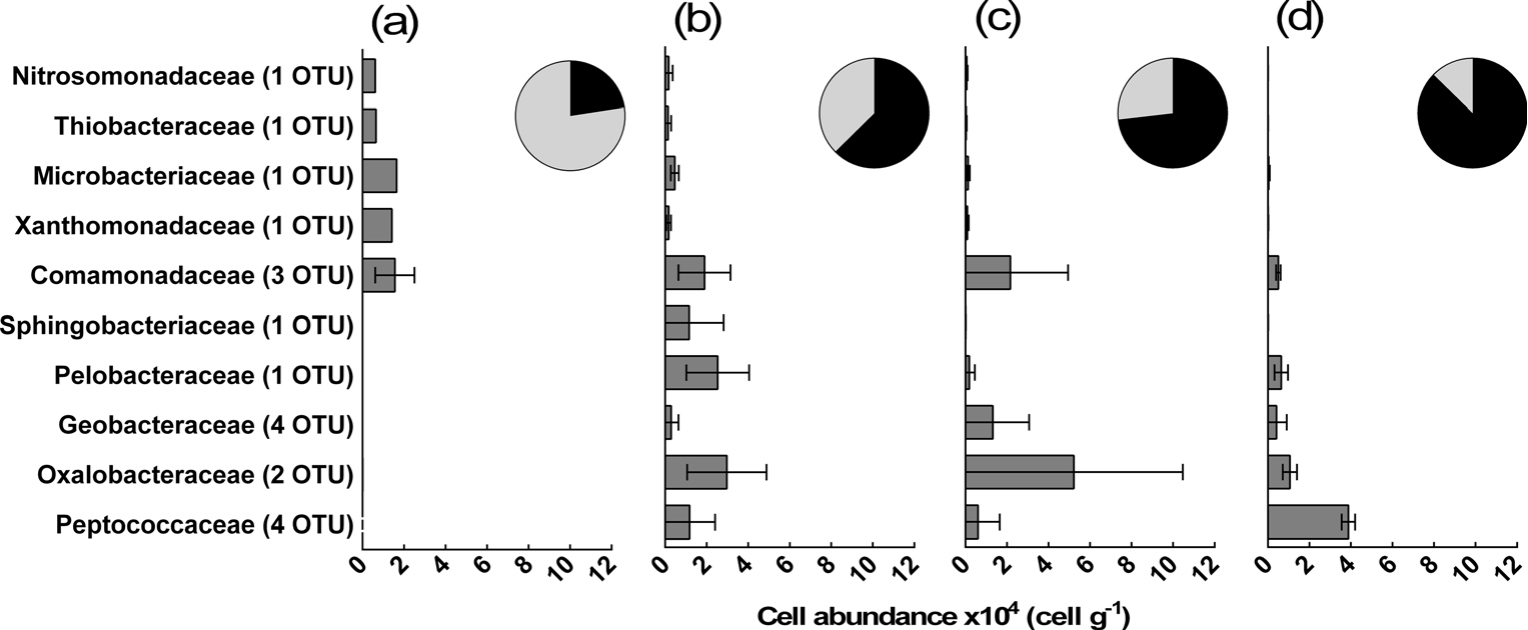
Estimated absolute abundance of cells associated with OTU (19) that contributed towards ≥1% of the dissimilarities found between CO_2_/H_2_-amended communities, within **a** T1; day 0, **b** T2; day 60, **c** T3; day 147 and **d** T5; day 371 sampled CO_2_/H_2_-amended communities. Estimations were calculated using qPCR analyses of 16S rRNA gene copy numbers in combination with reference data of 16S rRNA gene copy numbers per cell for each Greengenes OTU. Estimations are based on OTU grouped at the family level and the number of OTU associated with each family level group is displayed in *parentheses*. *Error bars* show the standard deviations. *Inset pie charts* depict the relative abundance of cells associated with these 19 OTU within each community, shown in *black*

The relative abundance of methane cycling organisms sequenced was low (0.10 ± 0.17%; Online Resource 2), contrary to previous studies of subglacial discharge samples [2, 5]. Methanogen-related amplicons that were closely related to *Methanomicrobiales* (two OTU) and *Methanosarcinales* (five OTU), including three OTU that were most closely related to anaerobic methanotrophic archaea (ANME) organisms, known also to be involved in sulphate-driven anaerobic oxidation of methane (AOM) [10], were identified in the day 0 and day 60 communities, which were consistent with previous studies of subglacial environments [5, 7, 11, 12] (Online Resource 2). Aerobic methylotrophs belonging to *Methylobacteriaceae* (four OTU) and *Methylocystaceae* (one OTU) were additionally identified (Online Resource 2). No methane cycling-related amplicons were detected within the final day 371 incubation.

The results of our methane cycling analysis are inconclusive; however, they inspire several hypotheses that warrant further investigation. First, we suggest that viable sulphate reducers and hydrogenotrophic methanogens are exported to the river delta from subglacial environments. Under the CO_2_/H_2_-amended experimental conditions, sulphate reducers thrive, while the concurrent loss of diversity and cell abundance suggests that a sizable portion of the original community is rendered unviable. Second, methane production is likely substrate limited, as it was only notably detected in incubations where hydrogenotrophic methanogenesis substrates were added. Based on the concurrent decrease in methane and sulphate concentrations, and the increasing relative abundance of *D. meridiei*, a sulphate-reducing bacterium, we propose that methanogens are out competed by sulphate-reducing bacteria, possibly as a result of the latter having a higher affinity to hydrogen [13]. Third, AOM by ANME species, which were identified within the incubations, although not on day 252, coupled with sulphate-reducing bacteria [14] such as *Desulfobulbaceae* and *Desulfobacteraceae* [15], may explain the methane sink that was observed between days 147 and 252, and this process may similarly occur in subglacial environments. Decreased partial pressures of hydrogen and elevated partial pressures of methane during the first months of incubation may have stimulated reverse methanogenesis, which likely mediates AOM [16]. We hypothesise that once sulphate is depleted, net methanogenesis may resume. Freshwater flux from the GrIS is predicted to increase over the coming decades [17], which will likely increase biomass transport from the subglacial environment [2]. This study finds that subglacial microbiota are a dynamic community that have the potential to contribute to the biogeochemical cycling of delta ecosystems, and we therefore suggest that any ecological significance will increase as climate warming continues.

## Electronic supplementary material

Online Resource 1 Supplementary methods. (PDF 148 kb)

Online Resource 2 Table showing the absolute abundance of amplicons related to potential methane cycling organisms. (PDF 125 kb)
