# Supplementary material for: Potential Activity of Subglacial Microbiota Transported to Anoxic River Delta Sediments
Source: Microb Ecol. 2017 Jan 9;74(1):6–9. doi: 10.1007/s00248-016-0926-2 (PMC5486838; doi:10.1007/s00248-016-0926-2)
Supplement: Supplementary file 1 — Supplementary methods. (PDF 148 kb) [file 248_2016_926_MOESM1_ESM.pdf]

Title: **Potential activity of subglacial microbiota transported to anoxic river delta sediments.**

Karen A. Cameron[1,2,3], Marek Stibal[1,2,4], Nikoline S. Olsen[1,2], Andreas B. Mikkelsen[2], Bo Elberling[2], Carsten S. Jacobsen[1,2,5]

[1]Department of Geochemistry, Geological Survey of Denmark and Greenland (GEUS), Øster Voldgade 10, DK-1350, Copenhagen, Denmark

[2]Center for Permafrost (CENPERM), University of Copenhagen, Øster Voldgade 10, DK-1350, Copenhagen, Denmark

[3]Institute of Biological, Environmental & Rural Sciences (IBERS), Aberystwyth University, Penglais, Aberystwyth, SY23 3FL, UK.

[4]Department of Ecology, Faculty of Science, Charles University, Viničná 7, 128 43, Prague, Czech Republic

[5]Department of Environmental Science, Aarhus University, Frederiksborgvej 399, DK-4000, Roskilde, Denmark

Corresponding author: Karen A. Cameron; E-mail: kac.geus@gmail.com, Telephone: +447764968773, Fax: na.

Journal: Microbial ecology

Type of paper: Notes and Short Communications

Online Resource 1:

Supplementary Methods

Site description and sampling

Sampling was performed in the river delta of the Watson River (67° 0' 07.1" N; 50° 43' 31.0" W) at the eastern end of Søndre Strømfjord, in southwest Greenland near Kangerlussuaq. A summary of the hydrological network that makes up the Watson River can be found within Cameron *et al*. (2016) [1]. Three sediment cores (25cm in length and 8 cm in diameter) were obtained on 26 September 2012 using ethanol-cleaned corers. The cores were packed into sterile Whirlpak bags and stored at 5 °C in the dark until analysis.

Incubation experiments

Long-term (371 day) anaerobic incubations were set up using sediment core material to determine the methanogenic potential of a subglacial-fed river delta system. A gas mixture of CO_2:H_2:N_2 (10:5:85%) was added to half of the incubations. Incubations without substrate additions were used as controls. Sediment core processing and steps thereafter were performed under sterile and anaerobic conditions. All unsterile equipment was autoclaved prior to use at 121°C and 15 psi for 30 minutes. Incubation vials were treated by submersion in 0.1 M HCl for 24 hours, rinsing in distilled and deionized water five times, then incubating at 300°C for 14 hours prior to storage in sealed autoclave bags. Incubation vials were then autoclaved on the day prior to setup. Sediment material was prepared by trimming and discarding ~3 cm from the ends of each core. The remaining material was mixed thoroughly, and composite subsamples (4 ± 0.02 g wet weight, equivalent to 2 ml) were randomly collected and transferred into sterile 25 ml

treated incubation vials. 18ml of anaerobic sterile distilled and deionized water, obtained by autoclaving distilled and deionized water then flushing it with purified nitrogen gas at a flow speed of 5.38 ml s^{-1} for at least 1 hour, was added to each incubation vial. The final headspace was 5 ml. Vials were sealed using butyl rubber stoppers and aluminium crimp caps. The headspaces of the CO_2/H_2 amended experiments were flushed with the gas mixture by leading 15 ml of gas through the water of the incubations. After removal of the needle, an additional volume of gas mixture (~ 3 ml) was added to obtain a pressure slightly above 1 atm. The headspaces of control experiments were flushed with purified N_2, following the same procedure as with the CO_2/H_2 amended incubations, to ensure anaerobic conditions. The study was set up so that five time points and two experimental conditions (control or CO_2/H_2 amended) were tested in triplicate, amounting to a total of 30 incubations. The time points for each experiment occurred between May 3, 2013, and May 9, 2014, and incubation periods are shown in Supplementary Table 1. Incubations incubated beyond day 0 were maintained at 2°C in the dark. Killed control incubations were additionally setup by autoclaving incubation vials, containing sediments and water, three times over eight days at 121°C and 15 psi for 30 minutes. Killed control incubations were set up for five time points and two experimental conditions, amounting to a total of 10 incubations. The headspace of these killed control incubations were flushed with purified N_2, using the same method as described previously. Despite repeated autoclaving of these incubations, biological activity was detected; therefore process rates from these killed controls have been omitted from this study. The persistence of microbes after stringent autoclaving has been reported previously [e.g. 2]. Regardless, 16S rRNA gene amplicons from killed control

communities were sequenced, and dominant amplicons that represented ≥ 1% relative abundance were used as markers of contamination, and were subsequently removed from all community profiles (see DNA extraction, quantification and sequence preparation).

	Control	CO_2/H_2
T1	0	0
T2	60	60
T3	115	147
T4	251	252
T5	371	371

Supplementary Table 1: Incubation periods in days for each experiment and time point

For methane concentration analysis, 1 ml of headspace gas was extracted from each incubation and was immediately injected into a gas chromatography flame ionization detector (SRI 8610C, Mikrolab Aarhus, Denmark). Methane concentrations were calculated relative to measurements of known standards. Concentrations in ppmv were converted into moles using the Ideal Gas Law equation. The limit of detection was calculated to be 1.90 x 10^{-10} mol and the limit of quantification was calculated to be 4.38 x 10^{-10} mol. Following gas analysis, samples for water chemistry analysis were obtained by opening the incubations, extracting water using a sterile 20 ml syringe, and filtering this through a 0.45µm Filtropur syringe filter (Sarstedt, Nümbrecht, Germany). Filtrate was collected into 20 ml plastic vials and water samples were kept in the dark at 5°C until analysis on a Dionex LC50-CD50 ion-chromatograph equipped with an IonPac AS14

column (Dionex, CA, USA). The limit of quantification was 0.05 mg L^{-1} (0.52 µmol L^{-1} or an absolute abundance of 0.00936 µmol within each incubation). Samples for community analysis were obtained by collecting ~0.50 g of sediment into a DNA-/Dnase/RNAse-free vial (Sarstedt, Nümbrecht, Germany). Community samples were snap-frozen in liquid nitrogen, and freeze-dried prior to storage at -80°C until further processing.

DNA extraction and quantification

Extraction of DNA from the incubation sediment samples was performed using the PowerSoil DNA Isolation Kit (MO BIO Laboratories, Carlsbad, CA, USA) according to the manufacturer's instructions with 0.25 g of sediment. Prokaryotic 16S rRNA genes from river sample DNA extracts were quantified using a qPCR set-up with primer pairs 341F (5'-CCTACGGGAGGCAGCAG-3') and 518R (5'-ATTACCGCGGCTGCTGG-3'; Muyzer *et al.*, 1993). Reaction mixtures (20 µl total) consisted of 1 µl of template DNA, 10 µl of SYBR Premix DimerEraser (TaKaRa, Japan) and 0.8 µl of the forward and reverse primers (10 pmol μl^{-1}). The cycle program was run using a CFX96 Touch real-time PCR detection system (Bio-Rad, CA, USA) at 95 °C for 30 s followed by 50 cycles of 95 °C for 30 s, 55 °C for 30 s and 72 °C for 30 s. The reaction was completed by a final 72 °C elongation step for 6 min and followed by high-resolution melt curve analysis in 0.5 °C increments from 55 to 98 °C. All qPCR reactions were performed in triplicate and were prepared under DNA free conditions in a pressurized clean-lab with a HEPA filtered air inlet and nightly UV-irradiation.

Amplicon sequencing preparation and analysis

Sequence library preparation, sequencing and downstream quality filtering was performed as described in Cameron *et al.* [3], with the exception that samples were rarefied to 2490 sequences per sample. All negative controls that were processed for sequencing had a final DNA concentration of ≤ 0.8 μg ml^{-1} and were therefore not sequenced. Operational taxonomic units (OTU) were defined as sequences that possessed ≥ 97% identity. In addition to removing chimeras and singletons, selected contaminant amplicons that made up ≥ 1% of the relative abundance of killed control communities, were filtered out of downstream analyses. These contaminants grouped into 135 OTU. An average of 20,793 amplicons per killed control sample were identified. In total, 167,294 contaminant selected amplicons were removed from the non-killed samples, equating to an average of 2,788 amplicons per sample. For sequence analysis, CatchAll was used to calculate parametric alpha diversity [4]. Untransformed Bray–Curtis resemblance, analysis of similarity (ANOSIM) and contributions of variables to similarity (SIMPER) were calculated using PRIMER-E version 6 (Plymouth, UK). Cell abundance data was generated using qPCR analyses in combination with the calculated number of 16S rRNA gene copy numbers per cell for each reference Greengenes OTU [5]. Amplicon data sets are available at The European Bioinformatics Institute under study accession number PRJEB14184. (http://www.ebi.ac.uk).

References:

1. Cameron KA, Stibal M, Hawkings JR, Mikkelsen AB, Telling J, Kohler TJ, Gözdereliler E, Zarsky JD, Wadham JL, Jacobsen CS (2016) Meltwater export of

prokaryotic cells from the Greenland ice sheet. Environ Microbiol. doi: 10.1111/1462-2920.13483

2. Yap JM, Goldsmith CE, Moore JE (2013) Integrity of bacterial genomic DNA after autoclaving: possible implications for horizontal gene transfer and clinical waste management. J Hosp Infect 83: 247-249. doi: 10.1016/j.jhin.2012.11.016

3. Cameron KA, Stibal M, Zarsky JD, Gözdereliler E, Schostag M, Jacobsen CS (2016) Supraglacial bacterial community structures vary across the Greenland ice sheet. FEMS Microbiol Ecol 92. doi: 10.1093/femsec/fiv164

4. Bunge J (2011) Estimating the number of species with CatchAll. Biocomputing: 121-130. doi:10.1142/9789814335058_0014

5. Langille MGI, Zaneveld J, Caporaso JG, McDonald D, Knights D, Reyes JA, Clemente JC, Burkepile DE, Vega Thurber RL, Knight R, Beiko RG, Huttenhower C (2013) Predictive functional profiling of microbial communities using 16S rRNA marker gene sequences. Nat Biotech 31: 814-821. doi: 10.1038/nbt.2676
