# Supplementary material for: Potential Activity of Subglacial Microbiota Transported to Anoxic River Delta Sediments
Source: Microb Ecol. 2017 Jan 9;74(1):6–9. doi: 10.1007/s00248-016-0926-2 (PMC5486838; doi:10.1007/s00248-016-0926-2)
Supplement: Supplementary file 2 — Table showing the absolute abundance of amplicons related to potential methane cycling organisms. (PDF 125 kb) [file 248_2016_926_MOESM2_ESM.pdf]

Title: **Potential activity of subglacial microbiota transported to anoxic river delta sediments.**

Karen A. Cameron[1,2,3], Marek Stibal[1,2,4], Nikoline S. Olsen[1,2], Andreas B. Mikkelsen[2], Bo Elberling[2], Carsten S. Jacobsen[1,2,5]

[1]Department of Geochemistry, Geological Survey of Denmark and Greenland (GEUS), Øster Voldgade 10, DK-1350, Copenhagen, Denmark

[2]Center for Permafrost (CENPERM), University of Copenhagen, Øster Voldgade 10, DK-1350, Copenhagen, Denmark

[3]Institute of Biological, Environmental & Rural Sciences (IBERS), Aberystwyth University, Penglais, Aberystwyth, SY23 3FL, UK.

[4]Department of Ecology, Faculty of Science, Charles University, Viničná 7, 128 43, Prague, Czech Republic

[5]Department of Environmental Science, Aarhus University, Frederiksborgvej 399,
DK-4000, Roskilde, Denmark

Corresponding author: Karen A. Cameron; E-mail: kac.geus@gmail.com, Telephone: +447764968773, Fax: na.

Journal: Microbial ecology

Type of paper: Notes and Short Communications

		Day 0			Day 60			Day 147			Day 371		
Family	GG OTU ID	#1	#2	#3	#1	#2	#3	#1	#2	#3	#1	#2	#3
Methanobacteriaceae	84255			96	46		65						
Methanomicrobiaceae	2260762	432											
ANME-2D	706603	29											
ANME-2D	805881						22						
ANME-2D	4378163			61									
Methanosaetaceae	794236		52										
Methanosarcinaceae	107074		52										
**Methanogen percentage**		**0.48**	**0.06**	**0.14**	**0.06**	**0.00**	**0.17**	**0.00**	**0.00**	**0.00**	**0.00**	**0.00**	**0.00**
Methylobacteriaceae	220715			16									
Methylobacteriaceae	68458	86											
Methylobacteriaceae	785526	17	188	19									
Methylobacteriaceae	4323871	14	26		15					7			
Methylocystaceae	1108830								20				
**Methylotroph percentage**		**0.12**	**0.12**	**0.03**	**0.02**	**0.00**	**0.00**	**0.00**	**0.04**	**0.02**	**0.00**	**0.00**	**0.00**
**Total percentage**		**0.60**	**0.17**	**0.18**	**0.07**	**0.00**	**0.17**	**0.00**	**0.04**	**0.02**	**0.00**	**0.00**	**0.00**

Online Resource 2: Absolute abundance (cells g^{-1}) of amplicons related to potential methane cycling organisms, described at the family level and with the Greengenes OTU identification number (GG OTU ID). Percentages are calculated based on the total cell abundance of each microcosm. Blank values indicate that amplicons were not detected.
